# Determinants of pelvic organ prolapse among adult gynecologic patients in Mekelle University Ayder Comprehensive Specialized Hospital, Tigray, Ethiopia: Case control study design

**DOI:** 10.1371/journal.pone.0322297

**Published:** 2025-04-24

**Authors:** Birhane Mekonen, Solomon Hintsa, Hailay Syum, Dawit Zenebe, Gebretsadik Kiros Lema, Ainom Shimondi, Redaie Girmay, Freweyni Fisshatsion, Goitom Girmay

**Affiliations:** 1 School of Medicine, College of Health Sciences, Aksum University, Aksum, Ethiopia; 2 School of Public Health, College of Health Sciences, Aksum University, Aksum, Ethiopia; 3 School of Public Health, College of Health Sciences, Mekelle University, Mekelle, Ethiopia; 4 School of Public Health, College of Health Sciences, Adigrat University, Adigrat, Ehiopia; 5 Tigray Region Health Bureau, Tigray, Ethiopia; 6 Department of Midwifery, College of Health Sciences, Aksum University, Aksum, Ethiopia; University of Oulu: Oulun Yliopisto, FINLAND

## Abstract

**Background:**

Pelvic organ prolapse is a major cause of morbidity among women in both high-income and low-income countries. Despite the severity of the problem, the risk factors associated with pelvic organ prolapse has been poorly understood in Ethiopia mainly in the study area. Hence, the purpose of this study was to identify determinants of pelvic organ prolapse among adult gynecologic patients at Mekelle University Ayder Comprehensive Specialized Hospital.

**Objective:**

To identify determinants of pelvic organ prolapse among adult gynecologic patients in Mekelle University Ayder Comprehensive Specialized Hospital, Tigray, Ethiopia, 2024.

**Methods:**

Hospital-based case-control study design was conducted from March 01, 2024 to May 30, 2024. All cases diagnosed with pelvic organ prolapse were enrolled in the study. Then, 4:1 control-to-cases ratio was applied. Data were entered into Epi Data version 4.6 and analyzed using SPSS version 21. Figures and tables were used for descriptive statistics. Variables with P-value less than 0.2 during binary logistic regression were labeled as candidates for multivariable logistic regression to identify independent predictors of pelvic organ prolapse at p-value < 0.05. The overall model fitness was checked by Hosmer Lemeshow at a P-value > 0.05. Finally, variables with P-value less than 0.05 and a 95% confidence interval of adjusted odds ratio were considered significant factors for the determinants of pelvic organ prolapse.

**Results:**

A total of 478 participants were recruited with a 100% response rate for both cases and controls. Low income (AOR=3.3; 95% CI: 1.1–9.7), vaginal tear (AOR=6.6; 95% CI: 2.5–17.6), menopausal status (AOR=9.2, 95% CI:2.3–37.4), body mass index <18 kg/m^2^ (AOR=6.3, 95% CI: 2.7–14.4), body mass index ≥25 (AOR= 5.6, 95% CI: 1.5–21.2) and chronic constipation (AOR=6.4, 95% CI: 2.9–13.9) were identified as determinants of pelvic organ prolapse.

**Conclusions:**

In this study, income of the participant, vaginal tear, menopausal status, body mass index (both underweight and overweight), and chronic constipation were factors found to be significantly associated with pelvic organ prolapse. Therefore, creating awareness about risk factors of pelvic organ prolapse, screening and early intervention, weight management program, and hormonal support is recommended.

## Introduction

Pelvic organ prolapse is defined as a descent of one or more of the following: the uterus (cervix), the anterior or posterior vaginal wall, or the vaginal apex (cuff scar or vaginal vault following hysterectomy) [1]. Pelvic organ prolapse has different classifications. Based on the pelvic organ prolapse–quantification (POP-Q) system, pelvic organ prolapse (POP) has different stages: stage 0 is no prolapse, stage 1 is the prolapse 1 cm above the hymen, stage 2 is the prolapse 1 cm or less away from the hymen, stage 3 is the prolapse over 1 cm below the hymen but at least 2 cm shorter than the total length of the vagina, and stage 4 is the entirety of the vagina everted itself 2 cm [2].

Pelvic organ prolapse (POP) affects approximately 40% of women globally, and as the population ages, they are predicted to become more prevalent [3]. According to a comprehensive research undertaken in countries with diverse economies, the burden of prolapse was substantially more significant in lower and middle-income nations (42.44%) than in higher-income ones (35.56%) [4].

According to studies conducted in Sub-Saharan Africa, the frequency of POP ranged from 12% to 64.6%. [5–7]. Based on a systematic review and meta-analysis conducted, the prevalence of pelvic organ prolapse in Ethiopia was 24.02% [8] whereas in Tigray was 1.2% [9].

Although not a life-threatening condition, pelvic organ prolapse can affect a woman’s quality of life by limiting physical, social, psychological, and sexual functions. A recent investigation revealed that pelvic organ prolapse (POP) diminishes the quality of life for women, affecting aspects such as their emotional well-being, sleep, interpersonal relationships, and social engagement [10]. Despite having a significant psychosocial influence on women's lives, a significant percentage of treatment delays occur because of societal stigmatization fears [11].

Several risk factors for the development of POP have been identified. Different studies showed that age, parity, body mass index (BMI), educational level, mode of delivery, prolonged labor, lifting heavy objects, chronic cough, and family history are some of the factors associated with pelvic organ prolapse[7,8,12–18]. Women in Tigray face unique socio-cultural, economic, and healthcare access challenges that influence their pelvic organ health [19]. Moreover, the recent conflict in the region has likely worsened these problems, resulting in a higher incidence of morbidity associated with pelvic organ prolapse [20].

Despite the severity of the problem, the risk factor associated with POP has been poorly understood in Ethiopia mainly in the study area in which there is limited research conducted on this problem. Therefore, this study aimed to identify the determinant of pelvic organ prolapse among adult gynecologic patients visiting Mekelle University Ayder Comprehensive Specialized Hospital, Tigray, north Ethiopia.

## Methods and material

### Study period and area

This was a hospital-based case-control study that was conducted from March 01,2024 to May 30, 2024 at Mekelle University Ayder Comprehensive Specialized Hospital (ACSH) in Mekelle town (783km North of Addis Ababa).

Mekelle is the capital city of the Tigray region and the largest city in northern Ethiopia, at a distance of 783 km from Addis Ababa. It has a population of more than 323,000 among these populations, there are 110,788 females, 104,758 males, 26,536 under five, 60,998 women of reproductive age (15–49 years), and 78,770 < 15 years age [21].

ACSH has a total capacity of about 500 inpatient beds in four major departments and other specialty units and 84 beds in the obstetrics & gynecology department. The obstetrics & gynecology department has 78 staff (20 gynecologists, 38 residents, and 20 midwifery) and is also used as a teaching hospital for the College of Health Sciences, Mekelle University. Residents are from year one to year four who are actively engaged in outpatient department, wards, and operation rooms performing their duties.

### Study design

A hospital-based case-control study design was applied.

### Eligibility criteria

#### Inclusion criteria.

**For controls:** All women aged 18 years and above free from POP but with some other gynecologic disease attending gynecologic OPD during the study period were included in the study.

**For cases:** All women aged 18 years and above diagnosed with POP attending gynecologic OPD during the study period were included in the study.

#### Exclusion criteria.

Critically ill and unable to give information women and those with mental problems from cases and controls were excluded.

### Operational definitions and measurements

Clients free of POP were taken as controls, and those who were diagnosed and confirmed to have POP by the physician on duty were taken as cases. Pelvic organ prolapse was evaluated and described using a standardized Pelvic Organ Prolapse Quantitative Examination tool [22].

#### Heavy load.

Those usual tasks involving lifting heavy objects/ doing extensive physical labor that strains the pelvic organs such as farming, carrying and marketing agricultural products, wood collection, and fetching water [23].

#### Light load.

Includes all tasks that do not involve the usual lifting of heavy objects/works that don’t require heavy force to strain the pelvic organ [23].

#### Chronic constipation.

Having difficulty passing feces which results in high straining of abdominal and pelvic muscles which occurs one or more times per month [24].

#### Chronic cough.

Having a cough for fourteen days duration resulting in high intra-abdominal pressure [16].

Income.

When the average income is less than 1.25 dollars per day is considered to be below the poverty line [25].

### Sample size determination

The sample size was calculated using Epi-info software version 7 using sample size determination for unmatched case-control studies. The parameters that were used to calculate sample size were a confidence level of 95%, power of 80%, a control-to-case ratio of 4: 1, a proportion of controls with exposure of 6.2%, a proportion of cases with exposure of 18.5%, and Odd Ratio of 3.1. It was calculated from the study conducted in Bahir Dar town, North West Ethiopia by taking BMI (< 18.5 kg/m2) as one of the main exposure variables for pelvic organ prolapse that provides the maximum sample size [16]. Therefore, it yields 87 cases and 347 controls. Adding a 10% non-response rate, the final sample size becomes 478 (96 cases and 382 controls).

### Sampling technique and procedure

On this research, we utilized a systematic random sampling method. First, we counted the total number of average control patients visiting the gynecology outpatient department (OPD). On average a minimum of 45 adult patients visit gynecology and obstetrics OPDs from Monday to Friday for treatment seek in each day. In total 225 patients seek the treatment per week,900 per month, and 2700 per 3 months.

Subsequently, all cases were included, and a control for each case was chosen using the systematic random sampling technique at every 7th patient. The selection of the first patient was carried out using a simple random sampling approach.

### Data collection method and procedure

The sources of data for this study were primary data. Data were collected using structured interviewer-administered questionnaires and chart reviews for the type and degree of prolapse were checked. The questionnaires were developed first in the English version and translated to the local language Tigrigna version and then translated back to English by different interpreters who were legal and fluent speakers of the languages to check for consistency. Three data collectors (Midwives working in maternal and child health clinic) and one supervisor (senior midwife) were trained on each item included in the questionnaires and get consent from the woman and collect data after the doctor took the history and to know the type and degree of prolapse data collectors were assisting the doctor during physical examination. Then collecting data until the required sample size was achieved, and then the principal investigator reviewed the questionnaires daily for completeness. Any questionnaires’ which was found incomplete were discarded and other eligible patients were asked.

### Data quality control

The questionnaires were prepared in English and translated into Tigrigna and back to English by independent language experts for consistency. A pretest was conducted by taking 5% of the total sample size (5 cases and 19 controls), pre-testing of the questionnaires in different settings was done at Adigrat General Hospital a week before the actual study and necessary modifications were made for the questionnaire according to the gap identified.

Three days of training were given to data collectors and supervisors on the content of the questionnaire and on how to collect data. The data collection process was strictly followed day to day by the supervisor and principal investigator and the collected data were checked for completeness and consistency every day. Before analysis, Simple frequencies were carried out to check data cleanness. Data cleanup and cross-checking were done.

### Data processing and statistical analysis

Collected data were entered into Epidata version 4.6 and exported to SPSS version 21 for further analysis. Data were entered, coded, and cleaned. Completeness and consistency were checked by running frequencies of each categorical variable. Normality was explored for continuous variables. Analyses of variables were made using descriptive statistics and unconditional binary logistic regression analysis to look into the predictors of pelvic organ prolapse. Variables with P-value less than 0.2 during binary logistic regression were labeled as candidates for multivariable logistic regression to identify independent predictors of pelvic organ prolapse at p-value < 0.05. Finally, variables with P-value less than 0.05 and a 95% confidence interval of adjusted odds ratio were considered significant factors for the determinants of pelvic organ prolapse.

The overall model fitness was checked by Hosmer Lemeshow at P-value >0.05 and it was fitted with p-value = 0.893. Multi-collinearity was also assessed at variance inflation factors (VIF<10).

### Ethics approval and consent to participate

Ethical clearance was obtained from the ethical review committee of Aksum University College of Health Science (IRB Number: 15/2024). Then a letter of cooperation was written from the School of Public Health to communicate with the concerned officials about the proposal. Permission was obtained from the respective officials. During the study, written informed consents and ascent were obtained from those greater or equal to 18-year-old participants included in the study. The right of participants to participate voluntarily and withdraw at any time during the study was respected. Confidentiality was maintained at all levels of the study. Data was kept secured at pass word locked personal computer.

## Results

### Socio-demographic characteristics of the study participants

A total of 478 women (96 cases and 382 controls) participated in the study which yielded a response rate of 100%. The median age of the respondents was 52 (interquartile range (IQR)19) and 36.0 (IQR 12) years among cases and controls, respectively.

Concerning the level of education 58(60.4%) of cases and 118 (30.9%) among controls were unable to read and write. Sixty-nine 69(71.9%) of cases and 172(45%)of controls were rural inhabitants. In the majority of the cases 85(88.5%) and controls 260 (68.1%) their income was less than or equal to 1.25 dollar per day (Table 1).

**Table 1:** Distribution of Socio-demographic characteristics among adult gynecologic patients in Mekelle University ACSH, Tigray, Ethiopia, 2024.

Variables	Categories	Cases (N = 96) Frequency (%)	Controls (N = 382) Frequency (%)
Age	≤49	36 (37.5)	320 (83.8)
	50-64	36 (37.5)	44 (11.5)
	≥65	24 (25)	18 (4.7)
Marital status	Married	68 (70.8)	290 (75.9)
	Divorced	13 (13.5)	54 (14.1)
	Widowed	14 (14.6)	30 (7.9)
	Separated	1(1)	8 (2.1)
Educational Level	Do not write or read	58 (60.4)	118 (30.9)
	Elementary	23 (24)	143 (37.4)
	Secondary	10 (10.4)	59 (15.4)
	College diploma or above	5 (5.2)	62 (16.2)
Occupation	Gov,t employee	15 (15.6)	63 (16.5)
	Private	4 (4.2)	138 (36.1)
	Housewife	51(53.1)	142 (37.2)
	Unemployed	1(1)	0 (0)
	Farmer	25 (26)	39 (10.2)
Religion	Orthodox Christian	94 (97.9)	351(91.9)
	Muslim	1(1)	21(5.5)
	Catholic	1(1)	4 (1)
	Protestant	0 (0)	6 (1.6)
Residence	Rural	69 (71.9)	172 (45)
	Urban	27 (28.1)	210 (55)
Income	≤1.25 $ per day	85 (88.5)	260 (68.1)
	>1.25 $ per day	11(11.5)	122 (31.9)

### Obstetric and gynecologic characteristics of the study participants

From the total POP cases 4 (4.2%) were stage one, 34 (35.4%) were stage two, 43 (44.8%) were stage three and 15 (15.6%) were stage four.

From the study participants more than half of 52(54.2%) cases and 141(36.9%) of controls had pregnancy experience greater or equal to four. Fifty-three (55.2%) of cases and 122(31.9%) controls gave birth at home. Fifty-one (53.1%) of cases and 133(34.8%) of controls had an average inter-pregnancy interval of ≤2 years. In the majority of cases, 79 (82.3%) and 367(96.1%) of controls gave birth vaginally to the last baby. Forty-four (45.8%) of cases and 54(14.1%) of controls had a history of vaginal tear.

Sixty-three (65.6%) of cases and 61(16%) of controls were in post-menopausal status. Seventy-one 71(74%) of cases and, 300(78.5%) of controls were married at the age of eighteen and above (Table 2).

**Table 2:** Distribution of obstetric and gynecologic characteristics among adult gynecologic patients in Mekelle University ACSH, Tigray, Ethiopia, 2024.

Variables	Categories	Cases (N = 96) Frequency (%)	Controls (N = 382) Frequency (%)
Number of pregnancy	≥4	52 (54.2)	141(36.9)
	<4	44 (45.8)	241(63.1)
Number of parity	≥4	49 (51)	130 (34)
	<4	47 (49)	252 (66)
Place of delivery (for the last birth)	Home	53 (55.2)	122 (31.9)
	Health institution	43 (44.8)	260 (68.1)
Return to work after delivery	<40 days	46 (47.9)	144 (37.7)
	≥42 days	50 (52.1)	238 (62.3)
Inter-pregnancy interval	≤2 years Two Three	51(53.1)	133 (34.8)
	>2 years	45(46.9)	249 (65.2)
Duration of labor (for the last birth)	≥8 hours	54 (56.3)	194 (50.8)
	<8 hours	42 (43.8)	188 (49.2)
Age at first delivery	<20 years	37(38.5)	132 (34.6)
	≥20 years	59 (61.5)	250 (65.4)
Sphincter damage	Yes	21(21.9)	35 (9.2)
	No	75 (78.1)	347 (90.8)
Vaginal tear (for the last birth)	Yes	44 (45.8)	54 (14.1)
	No	52 (54.2)	328 (85.9)
Carry heavy object	Yes	55 (57.3)	121 (31.7)
	No	41(42.7)	261(68.3)
Mode of delivery (the last baby)	SVD	79(82.3)	367(96.1)
	cesareansection	5(5.2)	15(3.9)
	instrumental	12(12.5)	0(0.0)
ANC follow up	Yes	64(66.7)	255(66.8)
	No	32(33.3)	127(33.2)
Episiotomy (during the last child birth)	Yes	39(40.6)	175(45.8)
	No	57(59.4)	207(54.2)
Ever used family planning	Yes	31(32.3)	206(53.9)
	No	65(67.7)	176(46.1)
Menopausal status	Postmenopausal	63(65.6)	61(16.0)
	premenopausal	33(34.4)	321(84.0)
Number of vaginal deliveries	≥4	46(47.9)	104(27.2)
	<4	50(52.1)	278(72.8)
History of abortion	Yes	13(13.5)	32(8.4)
	No	83(86.5)	350(91.6)
Age at first marriage	< 18 years	25(26)	82(21.5)
	≥18 years	71(74)	300(78.5)

NB. SVD=Spontaneous vaginal delivery, ANC=Ante Natal Care.

### Medical related factors and history of pelvic surgery

Of the study participants, 23 (24%) of cases and 56 (14.7%) of controls had a history of chronic cough. More than half of case 51(53.1%) and 41(10.7%) of controls had a history of chronic constipation. Among women involved in the study, 9(9.4%), 3(3.1%), and 2(2.1%) had a family history of POP, history of hypertension, and history of diabetic mellitus respectively.

Regarding participants’ BMI, 52(54.2%) of cases and 332(86.9%) of controls had BMI of 18.5–24.9 kg/m2 (Table 3).

**Table 3:** Medical-related factors and history of pelvic surgery among adult gynecologic patients in Mekelle University ACSH, Tigray, Ethiopia, 2024.

Variables	Categories	Case (N = 96) Frequency (%)	Control (N = 382) Frequency (%)
History of chronic cough	Yes	23(24)	56(14.7)
	No	73(76)	326(85.3)
Family history of POP	Yes	9(9.4)	3(0.8)
	No	87(90.6)	379(99.2)
BMI in [kg/m2]	<18.5	36(37.5)	33(8.6)
	≥25	8(8.3)	17(4.5)
	18.5-24.9	52(54.2)	332(86.9)
Chronic Constipation	Yes	50(52.1)	41(10.7)
	No	46(47.9)	341(89.3)
History of hypertension	Yes	3(3.1)	4(1.0)
	No	93(96.9)	377(99)
History of Diabetic Mellitus	Yes	2(2.1)	11(2.9)
	No	94(97.9)	371(97.1)
Ever smoke cigarette	Yes	0(0)	3(0.8)
	No	96(100)	379(99.2)
History of hysterectomy	Yes	1(1.0)	1(0.3)
	No	95(99.0)	381(99.7)
Previous prolapse surgery	Yes	9(9.4)	0(0.0)
	No	87(90.6)	382(100.0)

### Determinants of pelvic organ prolapse among adult gynecologic patients

The bi-variable logistic regression analysis showed that age, educational level, residence, income, number of pregnancies, number of parity, place of delivery, return to work after delivery, inter-pregnancy interval, sphincter damage, vaginal tear (for the last birth), carry heavy object, ever used family planning, menopausal status, number of vaginal deliveries, history of abortion, history of chronic cough, BMI in [kg/m2], chronic constipation were associated with pelvic organ prolapse at P-value ≤ 0.2.

After adjustment for possible potential confounding variables in the multivariable logistic regression analysis, income of the participant, vaginal tear (for the last baby), menopausal status, BMI, and chronic constipation were found to be significantly associated with pelvic organ prolapse at P-value < 0.05.

The odds of developing pelvic organ prolapse for patients with income ≤1.25 $ per day was 3.3 times more likely (AOR=3.3; 95% CI: 1.14-9.7) as compared to patients whose income was >1.25 $ per day. Likewise, patients who have vaginal tears were 6.6 times more likely (AOR=6.6; 95% CI%: 2.5-17.6) to develop pelvic organ prolapse as compared to their counterparts. The odds of pelvic organ prolapse for patients with postmenopausal status were 9.2 times (AOR=9.2, 95% CI: 2.3-37.4) more as compared to those who are in premenopausal status. The odds of pelvic organ prolapse were 6.3 times more (AOR=6.3, 95% CI: (2.7-14.4) among patients who had a BMI of <18 kg/m2 as compared to those with normal range of BMI (18.5-24.9) kg/m2. The odds of pelvic organ prolapse were 5.6 times more (AOR = 5.6, 95% CI: (1.5-21.2) among those who had BMI ≥25 kg/m2 as compared with those who had BMI of(18.5-24.9) kg/m2. Moreover, the odds of pelvic organ prolapse were 6.4 times more (AOR= 6.4, 95% CI: (2.9-13.9) among patients who had Chronic Constipation as compared with those who didn’t have chronic Constipation (Table 4).

**Table 4:** Bi-variable and multivariable logistic regression analysis of determinant of pelvic organ prolapse among adult gynecologic patients in Mekelle University ACSH, Tigray, Ethiopia, 2024.

variables	Categories	POP(N=478)		COR(95% CI)	P-Value	AOR (95% CI)	P-Value
		Cases (n = 96) Frequency(%)	Controls (n= 382) Frequency (%)				
Age	≤49	36(37.5)	320(83.8)	1		1	
	50-64	36(37.5)	44(11.5)	7.27(4.2-12.7)	.000	1.53(0.4-6.4)	.400
	≥65	24(25.0)	18(4.7)	11.85(5.9-23.9)	.000	1.45(0.3-7.6)	.517
Educational level	Donotwrite or read	58(60.4)	118(30.9)	6.09(2.3-16.0)	.000	0.9(0.2-4.3)	.924
	Elementary	23(24.0)	143(37.4)	2.0(0.7-5.48)	.181	0.4(0.1-2.0)	.281
	Secondary	10(10.4)	59(15.4)	2.1(0.67-6.51)	.198	0.7(0.2-3.6)	.696
	College diploma	5(5.2)	62(16.2)	1		1	
Residence	Rural	69(71.9)	172(45.0)	3.1(1.9-5.1)	.000	1.3(0.6-3.1)	.496
	Urban	27(28.1)	210(55.0)	1		1	
Income	≤1.25 $ per day	85(88.5)	260(68.1)	3.6(1.9-7.0)	.000	**3.3(1.1-9.7)** *	.027
	>1.25 $ per day	11(11.5)	122(31.9)	1		1	
Number of pregnancy	≥4	52(54.2)	141(36.9)	2.0(1.3-3.2)	.002	2.4(0.4-13.3)	.316
	<4	44(45.8)	241(63.1)	1		1	
Number of parity	≥4	49(51.0)	130(34.0)	2.0(1.3-3.2)	.002	0.2(0.03-1.5)	.126
	<4	47(49.0)	252(66.0)	1		1	
Place of delivery	Home	53(55.2)	122(31.9)	2.6(1.6-4.1)	.000	0.9(.3-2.6)	.833
	Health institution	43(44.8)	260(68.1)	1		1	
Return to work after delivery	<42 days	46(47.9)	144(37.7)	1.5(0.96-2.38)	.068	1.0(0.5-2.2)	.929
	≥42days	50(52.1)	238(62.3)	1		1	
Inter-pregnancy interval	≤2 years Two Three	51(53.1)	133(34.8)	2.1(1.3-3.3)	.001	1.70 (0.9-3.4)	.126
	>2 years	45(46.9)	249(65.2)	1		1	
Sphincter damage	Yes	21(21.9)	35(9.2)	2.8(1.5-5.0)	.001	0.7(0.2-2.3)	.593
	No	75(78.1)	347(90.8)	1		1	
Vaginal tear (for the last birth)	Yes	44(45.8)	54(14.1)	5.1(3.1-8.4)	.000	**6.6(2.5-17.6)** **	.000
	No	52(54.2)	328(85.9)	1		1	
Carry heavy object	Yes	55(57.3)	121(31.7)	2.9(1.8-4.6)	.000	1.8(0.9-3.7)	.109
	No	41(42.7)	261(68.3)	1		1	
Ever used family planning	Yes	31(32.3)	206(53.9)	1		1	
	No	65(67.7)	176(46.1)	2.5(1.5-3.9)	.000	0.9(0.4-2.1)	.914
Menopausal status	Postmenopausal	63(65.6)	61(16.0)	10(6.0-16.6)	.000	**9.2(2.3-37.4)** *	.005
	premenopausal	33(34.4)	321(84.0)	1		1	
Number of vaginal deliveries	≥4	46(47.9)	104(27.2)	2.5(1.6-3.9)	.000	1.25(0.4-4.3)	.726
	<4	50(52.1)	278(72.8)	1		1	
History of abortion	Yes	13(13.5)	32(8.4)	1.7(0.9-3.40)	.125	2.0(0.8-5.5)	.155
	No	83(86.5)	350(91.6)	1		1	
History of chronic cough	Yes	23(24.0)	56(14.7)	1.8(1.1-3.2)	.030	0.7(0.3-1.7)	.458
	No	73(76.0)	326(85.3)	1		1	
BMI in [kg/m2]	<18.5	36(37.5)	33(8.6)	6.1(4.0-12.1)	.000	**6.3(2.7-14.4)** **	.000
	≥25	8(8.3)	17(4.5)	3(1.2-7.3)	.015	**5.6(1.5-21.2)** *	.011
	18.5-24.9	52(54.2)	332(86.9)	1		1	
Chronic Constipation	Yes	50(52.1)	41(10.7)	9(5.40-15.1)	.000	**6.4(2.9-13.9)** **	.000
	No	46(47.9)	341(89.3)	1		1	

NB. Statistically significant at *=(p<0.05), ** =(p<0.001); 1: reference group; AOR = Adjusted Odds Ratio, COR = Crude Odds Ratio, C.I = Confidence Interval, BMI=Body Mass Index, $=Dollar

## Discussion

This study aimed to identify determinants of pelvic organ prolapse among adult gynecologic patients in Ayder Comprehensive Specialized Hospital and revealed that low income of the participant, vaginal tear (for the last baby), menopausal status, BMI, and chronic constipation were found significantly associated with pelvic organ prolapse.

The likelihood of developing pelvic organ prolapse for patients with income ≤1.25 $ per day was 3.3 times more likely as compared to patients whose income was >1.25 $ per day. This finding is supported by the case-control studies done in public hospitals in southern Ethiopia and multicenter cross-sectional studies done in the United States of America [26,27]. The possible explanation is that women with higher incomes are more likely to receive better health care, seek treatment on time, prefer occupations, get adequate nutrition and someone to support them.

Patients who have vaginal tears were 6.6 times more likely to develop pelvic organ prolapse as compared to their counterparts. This is in line with a study done in southern Ethiopia [26] Bahirdar [16] and Uganda [28]. This is owing to an injury to neuro-vasculature, disruption of the muscles, perineal membrane, ligaments, and other connective tissues of the pelvic floor that support and maintain female pelvic structures in an anatomical position [29].

The odds of pelvic organ prolapse for patients with postmenopausal status were 9.2 times more as compared to those who are in premenopausal status. This finding is supported by a cross-sectional study done in Nigeria [18]. During menopause, the lack of estrogen causes atrophy of the genital tract musculature and reduces the strength of the connective tissue supporting it leading to the development of POP.

Like studies from Bahir Dar, Nekemte western Ethiopia, Benchimaji zone [16,17,30], underweight (BMI <18.5 kg/m2) was found to be a determinant of UVP in this study. This is due to the possibility of micronutrient deficiencies which are necessary for connective tissue strength.

On the other hand, this study also showed that obesity was a risk for POP. The findings of this study revealed that patients who were overweight developed POP 5.6 times more likely than patients with normal weight. The possible justification for this could be increased intra-abdominal pressure that causes the weakening of pelvic floor muscles and fascia in obese patients. This is in line with the findings of a case-control study in Akista northern Ethiopia and Arbaminch southern Ethiopia [13,31].

In addition, the odds of pelvic organ prolapse were 6.4 times higher among patients who had chronic constipation as compared with those who didn’t have chronic Constipation. This finding is consistent with the cross-sectional study in Jimma and the case-control study in Wolaita sedo [12,23]. A possible explanation is women with constipation often experience chronic straining during bowel movement. Hence this increased intra-abdominal pressure can weaken the pelvic floor muscles and connective tissues over time, making prolapse more likely.

## Conclusions

In this study income of the participant, vaginal tear, menopausal status, body mass index (both underweight and overweight), and chronic constipation were factors found to be significantly associated with pelvic organ prolapse. These findings underscore the multifaceted nature of POP, suggesting that it is not solely a result of anatomical changes but is also influenced by socioeconomic and lifestyle factors. Implement educational programs aimed at raising awareness about POP risk factors, and preventive measures. Focus on communities with low income to enhance understanding and encourage early reporting of symptoms. Establish routine screening for POP in gynecological clinics, particularly for women with identified risk factors such as low income, history of vaginal tears, and chronic constipation. Early diagnosis can lead to timely interventions and improved outcomes. Develop community-based weight management and nutrition programs to address obesity and underweight issues, as both extremes of BMI are associated with increased POP risk. Promote healthy lifestyle choices to support pelvic health. Increasing dietary fiber, adequate fluid intake and laxatives can help alleviate constipation by making bowel movements easier and reducing the need for straining. Provide resources and support for women experiencing menopause, including counseling on hormonal therapies and pelvic floor exercises, which may help mitigate the effects of hormonal changes on pelvic support structures. Longitudinal studies are recommended to better understand the causal relationships and progression of POP over time.

## Strengths and limitations of the study

### Strengths

#### Clinical diagnosis.

Emphasize that the study's outcome variable was based on physician diagnosis, ensuring accuracy and reliability in identifying cases of POP. This strengthens the validity of the findings.

#### Comprehensive data collection.

Highlight the thoroughness of the data collection process, which included a wide range of socio-demographic, obstetric, gynecologic, and medical history factors, allowing for a holistic view of the determinants of POP.

#### High response rate.

Note the 100% response rate, which enhances the representativeness of the sample and reduces selection bias.

### Limitations

It has also some limitations like recall bias because patients may forget their experiences or health-related events before developing POP. Using an unmatched study design was also another limitation. In addition, the study was conducted at the hospital level, so we did not include patients with less severe symptoms.
